# Influence of HIV infection on cognition and overall intelligence in HIV-infected individuals: advances and perspectives

**DOI:** 10.3389/fnbeh.2023.1261784

**Published:** 2023-10-26

**Authors:** Silvere D. Zaongo, Vijay Harypursat, Farooq Rashid, Désiré Lucien Dahourou, Abdoul-Salam Ouedraogo, Yaokai Chen

**Affiliations:** ^1^Department of Infectious Diseases, Chongqing Public Health Medical Center, Chongqing, China; ^2^Département Biomédical/Santé Publique, Institut de Recherche en Sciences de la Santé/CNRST, Ouagadougou, Burkina Faso; ^3^Centre Muraz, Bobo-Dioulasso, Burkina Faso; ^4^Department of Bacteriology and Virology, Souro Sanou University Hospital, Bobo-Dioulasso, Burkina Faso

**Keywords:** HIV, intelligence, intelligence quotient, brain, neuron, gut

## Abstract

It is now well understood that HIV-positive individuals, even those under effective ART, tend to develop a spectrum of cognitive, motor, and/or mood conditions which are contemporarily referred to as HIV-associated neurocognitive disorder (HAND), and which is directly related to HIV-1 infection and HIV-1 replication in the central nervous system (CNS). As HAND is known to induce difficulties associated with attention, concentration, and memory, it is thus legitimate and pertinent to speculate upon the possibility that HIV infection may well influence human cognition and intelligence. We therefore propose herein to review the concept of intelligence, the concept of cells of intelligence, the influence of HIV on these particular cells, and the evidence pointing to differences in observed intelligence quotient (IQ) scores between HIV-positive and HIV-negative individuals. Additionally, cumulative research evidence continues to draw attention to the influence of the gut on human intelligence. Up to now, although it is known that HIV infection profoundly alters both the composition and diversity of the gut microbiota and the structural integrity of the gut, the influence of the gut on intelligence in the context of HIV infection remains poorly described. As such, we also provide herein a review of the different ways in which HIV may influence human intelligence via the gut-brain axis. Finally, we provide a discourse on perspectives related to HIV and human intelligence which may assist in generating more robust evidence with respect to this issue in future studies. Our aim is to provide insightful knowledge for the identification of novel areas of investigation, in order to reveal and explain some of the enigmas related to HIV infection.

## Introduction

Despite decades of massive financial and intellectual investment, human immunodeficiency virus (HIV) infection remains a global public health burden today. Indeed, although modern antiretroviral therapy (ART) efficiently suppresses HIV-1 replication, thereby allowing HIV-infected individuals to live a relatively normal life (i.e., improved health outcomes and a consequent life expectancy comparable to HIV negative individuals), there remains as yet no cure for HIV (Zaongo et al., 2020, 2021, 2022a). Regrettably, ART must necessarily be a lifelong therapeutic necessity due to the dormant presence of HIV provirus within the genomes of infected cells, which leads inexorably to HIV viral rebound upon ART cessation (Ho et al., 2013). This represents a significant psychological, physical, and financial burden for patients (Phillips A. N. et al., 2016) who also must contend with ART-related toxicity directed toward vital organs such as the kidneys (Venter et al., 2018), the liver (Taramasso et al., 2019), the heart (Garg et al., 2013), the brain, and the central and peripheral nervous systems (Treisman and Soudry, 2016). The infected cells harboring latent HIV provirus, also referred to as the HIV reservoir, therefore represent the major challenge to curing HIV infection (Siliciano and Greene, 2011). In general, the HIV reservoir comprises many types of cells originating from different organs (lymph nodes and spleen, bone marrow, thymus, liver, gastrointestinal tract, among others; Wong and Yukl, 2016). Investigations over recent years have observed the importance of the brain as a potential reservoir for persistent HIV infection (Saylor et al., 2016).

It is well known that HIV is a neurotropic virus which affects the brain, and has the potential to impair the integrity of the blood–brain barrier (BBB; Atluri et al., 2015; Zhang et al., 2015; McRae, 2016; Patel et al., 2017; Rahimy et al., 2017; Bertrand et al., 2019; Wallet et al., 2019). As such, viral proteins interfere with endothelial cells by modifying the small unit of GTPases and ERK1/2, and by increasing the generation of reactive oxygen species (ROS; Toborek et al., 2003; Pu et al., 2005; Zhong et al., 2012). In addition to ROS, the elevated levels of proinflammatory cytokines [Tumor necrosis factor (TNF)-α, Interleukin (IL)-1β] and C-reactive protein may encourage and exacerbate the brain inflammation (Brabers and Nottet, 2006; Ross et al., 2009; Younas et al., 2016). Therefore, a likely consequence of the preceding changes is to potentially provoke an undiscriminatingly leaky BBB, which impacts the function of endothelial cells and, more seriously, induces brain damage resulting from the destruction of astrocytes and pericytes (Ahmed et al., 2018).

One of the major consequences of infection of brain tissue by HIV is the emergence of a spectrum of cognitive, motor, and/or mood problems, collectively referred to as HIV-associated neurocognitive disorder (HAND). In general, HAND is characterized by typical symptoms, such as (i) difficulties with attention, concentration, and memory, (ii) loss of motivation, (iii) irritability, (iv) depression, and (iv) slow movements (UCSF Weill Institute, 2022). In 2010, it was reported that up to 30%–50% of people living with HIV were afflicted by HAND, most of whom had mild forms of HAND (Heaton et al., 2010). Recent data from a meta-analysis by Wang Y. et al. (2020) suggests that among HIV-infected individuals, there are roughly 42.6% suffering from HAND, with 88% of cases representing mild disease. These observations indicate that HAND remains highly prevalent in this era of effective ART, particularly in sub-Saharan Africa and Latin America. Furthermore, the proportion of individuals with HAND has been observed to correlate inversely with levels of income, CD4+ T-cell counts, and ART administration (Wang Y. et al., 2020).

With established evidence indicating that HAND induces difficulties with attention, concentration, and memory, it is quite pertinent and appropriate to speculate on the influence of HIV infection on intelligence quotient (IQ) scores of HIV-infected patients. One study conducted in Peru in 2012 concluded that HIV positive children achieve lower IQ scores in comparison to HIV negative children (Martinez et al., 2012). Van den Hof et al. (2019) demonstrated that perinatally HIV-infected (PHIV) children have significantly lower IQs compared than HIV-uninfected controls. As the brain becomes infected and some cognitive functions consequently deteriorate, HIV infection may potentially thereby influence the level of intelligence of HIV-infected individuals. However, human intelligence remains challenging to define, although it is acknowledged that intelligence refers to a person’s inherent capacity to memorize, plan ahead, reason, and solve problems (Goriounova and Mansvelder, 2019). The effects and influence of HIV infection on the human brain demands rational discussion with respect to the potential influence of HIV infection on human cognition and overall intelligence. Securing a less nebulous picture of the preceding scenario would require novel areas of investigation, leading to the implementation of more appropriate and relevant interventions in order to improve the brain health of patients living with HIV infection. In this article, we discuss the influence of HIV infection on the neurobiological basis of human intelligence. Specifically, we define human intelligence from a neurobiological perspective, discuss the concept of cells of intelligence, explore the effects of HIV infection on these particular cells of intelligence, and review research evidence which establishes a clear relationship between HIV infection and IQ score reduction. We also review the influence of the gut on intelligence through the gut-brain axis. This is motivated by the pivotal role that is now known to be played by the gut in HIV immunopathogenesis. Indeed, the gut is the earliest target of HIV infection, and harbors the major fraction of the HIV reservoir (Wong and Yukl, 2016). Additionally, HIV disrupts gut-associated lymphoid tissue to induce the leaky gut syndrome, local and systemic inflammation, and immune activation (Chun et al., 2008; Sereti et al., 2017). These effects exerted by HIV are likely to influence inherent gut function and the particular interactions of the gut with the brain. As such, Rich et al. (2020) have reported that in HIV infected individuals, the microbiome-gut-brain axis is likely to potentially mediate the emergence of HIV-associated neurocognitive disorders. Earlier, Al-Asmakh et al. (2012) have also indicated that gut microbial communities may modulate brain development and functions. In light of the findings of the preceding publications, the gut-brain axis may well be seen as another potential factor influencing human intelligence, and therefore the influence of HIV on the gut-brain axis deserves diligent examination. Thus, after an overall presentation on how the gut may influence and modulate brain development and functions, we specifically discuss the mechanisms whereby HIV may influence human intelligence through its interactions with the gut via the gut-brain axis. Lastly, we provide a discourse on perspectives which may assist in garnering more robust investigative evidence for HIV-related intelligence studies in future research endeavors.

## Definition of intelligence

Intelligence is a particularly difficult concept to define. Intuitively, “intelligence” is accepted as the attribute which, in humans, helps us to memorize, plan ahead, reason, solve problems, and make rational decisions (Goriounova and Mansvelder, 2019). However, defining intelligence is not as simple as it seems. According to Sternberg (2012), intelligence is defined as the ability to learn from experience and to adapt to and mold different environments. It is human intelligence and the capacity for deliberate and rational cognition that have ultimately helped and enabled humans to survive and thrive in the prevailing environment, from the first iteration of primitive *Homo sapiens* to modern humans living in the fast-paced, modern, and technologically advanced world in which we now live and thrive (Ko, 2016). In light of difficulties to define intelligence, specific cognitive tests have been designed to measure human performance in different cognitive domains, such as processing speed and language (Goriounova and Mansvelder, 2019). However, intelligence and cognition are different. While intelligence refers to an ability to learn from experience and to adapt and mold different environments, cognition is a synthetic mental process whereby a person acquires knowledge and comprehension (American Psychological Association, 2023). The results of these cognitive tests are reported to strongly correlate with capabilities referred to as general intelligence or “Spearman’s *g*” (Spearman, 1904). For example, the Wechsler adult intelligent scale (WAIS), one of the most commonly used tests to estimate Spearman’s *g* in contemporary times, combines the results of multiple cognitive tests into one measurement, referred to as the full-scale intelligence quotient (IQ) score (Goriounova and Mansvelder, 2019). However, criticisms have been made regarding the ability to measure the entire spectrum of human intelligence via a single number (referred to as the IQ score). Although the preceding criticisms may be legitimate (as IQ scores may constitute a reductionist approach in exploring the entire spectrum of human intelligence), IQ testing remains valid and relevant due to its robust correlation with life outcomes, including socioeconomic status and cognitive ability, even when measured early on in life (Foverskov et al., 2019).

It has been reported that intelligence is a very stable trait in a particular individual from young to old age (Goriounova and Mansvelder, 2019). Indeed, in one large longitudinal study in English children, a correlation level of 0.81 was observed between measured intelligence at 11 years of age and scores on national tests of educational achievement 5 years later (Deary et al., 2007). In another study, it has been shown that a single test of general intelligence taken at age 11 correlates highly (0.54 and 0.67 when corrected) with results of the equivalent test at the age of 90 (Deary et al., 2013). Additionally, some investigative outcomes suggest that intelligence is also heritable. Posthuma et al. have observed from studies of twins that heritability of intelligence is extraordinarily large, in the range of 50% to 80%, and even extends to 86% for verbal IQ (Posthuma et al., 2001). Thus, Plomin and Deary have postulated that human intelligence may be one of the most heritable human behavioral traits (Plomin and Deary, 2015).

**Figure 1:**
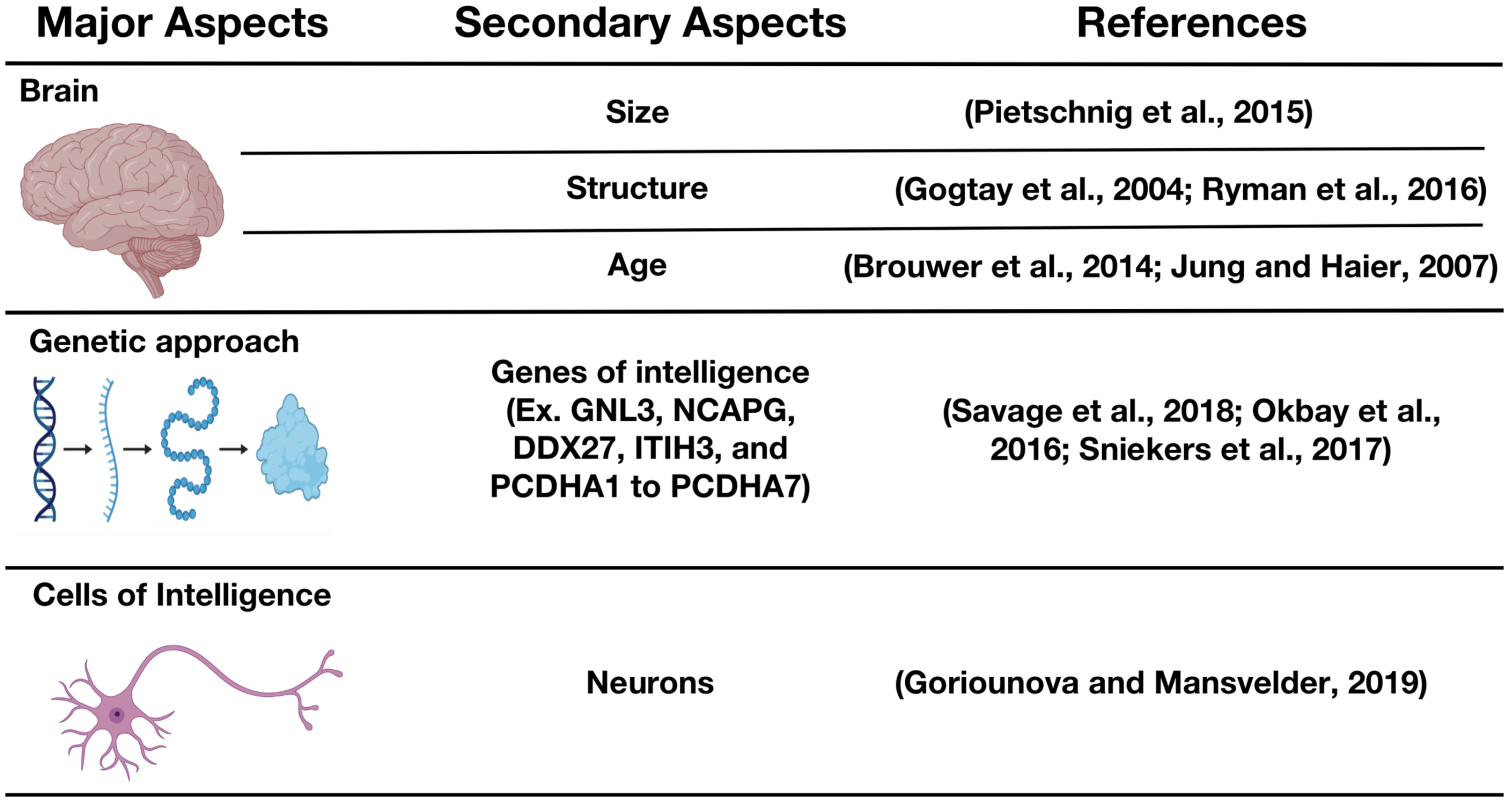
Biological basis of intelligence. GNL3, Guanine nucleotide-binding protein-like 3; NCAPG, non-SMC condensin I complex subunit G; DDX27, DEAD (Asp-Glu-Ala-Asp) box polypeptide 27; ITIH3, Inter-alpha-trypsin inhibitor heavy chain H3; PCDHA-(1to 7), Protocadherin alpha-(1 to 7) (Gogtay et al., 2004; Jung and Haier, 2007; Brouwer et al., 2014; Pietschnig et al., 2015; Okbay et al., 2016; Ryman et al., 2016; Sniekers et al., 2017; Savage et al., 2018; Goriounova and Mansvelder, 2019).

In the quest to address concerns with respect to the origins of human intelligence or, simply put, the question as to precisely “what makes some people smarter than others,” we have discovered that one hypothesis that has been used rather ubiquitously over the past century has assumed that smarter (or more intelligent) people have comparatively bigger brains. This hypothesis has been put to test in many studies, and overall outcomes reveal that a bigger brain volume is, indeed, associated with higher intelligence (McDaniel, 2005; Pietschnig et al., 2015). However, as proposed by Goriounova and Mansvelder, human intelligence may be more comprehensively considered from three distinct major perspectives (Figure 1), which are: (i) the whole-brain perspective (mainly covering brain size and structure), (ii) the genetic approach to intelligence (referring to genes of intelligence, their polymorphisms, and their roles in cell–cell interactions), and (iii) cells of intelligence (mainly neurons and their interconnections; Goriounova and Mansvelder, 2019). We will focus our discussion on the cells associated with intelligence, the potential influence of HIV infection on these cells as reported in contemporary literature, and we will, lastly, discuss research evidence showing IQ scoring differences between HIV-positive and HIV-negative individuals.

## Cells of intelligence

Since 1893, when Cajal postulated his “neuron doctrine” (Cajal, 1893), it has been acknowledged and understood that the fundamental basis of human intelligence must lie in neurons or networks of neurons. However, the macroscopic attributes of the brain and the genes potentially involved in cognition remain the most explored subjects in this field, thus exposing a large knowledge gap at the cellular level (Goriounova and Mansvelder, 2019). The human mind functions via the co-ordinated activity of its 86 billion neurons (Herculano-Houzel, 2012), and their vast network of neural connections. Thus, this neural network which drives centers for coding, processing, and storage of information in the brain ultimately gives rise to cognition, as proposed by Salinas and Sejnowski (2001). From this basis, and given the colossal number of interneuronal connections present (Drachman, 2005), even minor changes in the efficiency of information processing by neurons may result in meaningful differences in cognitive capacity.

Studies (Rogalski et al., 2013; Gefen et al., 2015) have shown that the cortex, which is responsible for thinking, decision-making, and memory, remains much thicker and shrinks much more slowly in younger individuals than in older people in their 6th and 7th decades of life. The authors of the preceding studies have suggested that the overall reduction in cognitive functions seen in older individuals occurs as a result of the observed magnetic resonance imaging (MRI) changes seen in older persons. These findings can be considered as initial fundamental steps in the understanding of intelligence, and of the regions of the brain which are most related to intelligence. The human brain is made up of billions of cells, and includes neurons and glial cells, both of which have an unknown number of subtypes (Herculano-Houzel, 2012). Notably, it has been demonstrated that expression of genes associated with higher intelligence accumulates in cortical pyramidal neurons (Savage et al., 2018; Coleman et al., 2019). The brain samples used in the preceding studies were harvested from the temporal cortices of test patients, mainly in order to spare primary sensory and language functions in the test patients (Goriounova and Mansvelder, 2019). These findings prompt the following question: what is the role of pyramidal neurons in human intelligence? First, one study by Mohan et al. has shown that compared to rodents and macaques, human layer 2/3 pyramidal cells have threefold larger and more complex dendrites (i.e., dendrites that inherently possess more elaborate and labyrinthine arborization patterns; Mohan et al., 2015). In an earlier study, DeFelipe et al. indicated that large human dendrites receive double the number of synapses compared to rodent pyramidal neurons (DeFelipe et al., 2002). Also, Testa-Silva et al. (2014) have reported that in addition to structural differences to pyramidal neurons in other species, human pyramidal neurons may also display unique functional properties. They have noted that human excitatory synapses recover three to four times faster than synapses in the rodent cortex, have faster action potentials, and transfer information up to nine times more rapidly than murine synapses. Regarding these differences across species, it is likely that adaptative pressure may have helped humans to develop pyramidal neurons that are able to perform cognitive functions. Interestingly, one study by Goriounova et al. has demonstrated that differences in human pyramidal neuronal structure and function can be linked to intelligence scores, and to the anatomical structure of the temporal lobes (Goriounova et al., 2018). The preceding authors have observed for the first time in their study that higher IQ scores are associated with larger dendrites, faster action potentials during neuronal activity, and more efficient information tracking in the pyramidal neurons of the temporal cortex. Recently, one research team (Nassif et al., 2022) observed that neurons in the cerebral cortex have direct connections to another key memory center, the hippocampus, and the structural and functional integrity of these neurons is essential for memory and learning. Thomaidis et al. (2010) observed that HIV patients without neuroimaging abnormalities have similar cognitive development compared to their healthy peers. However, patients with neuroimaging abnormalities have been observed to have lower IQ scores (*p* < 0.05; see Table 1). In light of the major role played by neurons in human intelligence, a more complete understanding of IQ decline during HIV infection requires a much broader and more comprehensive appreciation of the effects of HIV on neurons.

**Table 1:** Contemporary literature evaluating intelligence quotient scores during HIV infection.

Authors (publication year)	Region	Subjects (sample size)	Age median or mean	Family income	Approach	IQ scores
Martinez et al. (2012)	Peru	HIV+ Children (28)	N/A	N/A	Wechsler intelligence scale	Full-scale IQ = 84.6 vs. 91.7
		HIV− Children (18)	N/A			
Kerr et al. (2014)	Thailand and Cambodia	HEU (160)	7.4	57% Low income	Wechsler intelligence scale	Verbal IQ = 81.1 vs. 87.2
		HUU (167)	7.7	34% Low income		Full-scale IQ = 85.2 vs. 90.3
Cohen et al. (2015)	The Netherlands	PHIV+ children (35)	13.8	N/A	Wechsler intelligence scale	Verbal IQ = 77.9 vs. 89.6
		HIV− children (37)	12.1			Performance IQ = 78.1 vs. 86.5
						Full-scale IQ = 76 vs. 87.5
Sunmonu et al. (2015)	Nigeria	HIV+ adults (58)	35.9	N/A	Wechsler intelligence scale	Significantly lower mean z-scores for verbal and performance IQ
		HIV− adults (50)	35.4			
Van den Hof et al. (2019)	The Netherlands and Sub-Saharan Africa	HIV+ children (14)	10.45	N/A	Wechsler intelligence scale	Full-scale IQ = 81.4 vs. 97
		HIV− Children (15)	10.69			
Ananworanich et al. (2014)^*^	Thailand	ART-Naïve (67)	9	39% Low income	Wechsler intelligence scale	Full-scale IQ = 75
						Verbal IQ = 73
						Performance IQ = 80
Patel et al. (2019)^**^	Thailand	PHIV+ (165)	8.2	40.6% Low income	Wechsler intelligence scale	Verbal IQ declined as age increased and household income decreased
Murthy et al. (2018)	India	PHIV (42)	10.71	N/A	Wechsler intelligence scale	Verbal IQ = 71.3 vs. 90
						Full-scale IQ = 79.8 vs. 96.6
		HIV− (40)	11.53			Performance IQ = 93.4 vs. 104.3
Thomaidis et al. (2010)	Greece	PHIV without NA (15)	12.35	N/A	Wechsler intelligence scale	Full-scale IQ = 82.4 vs. 58.8 vs.78
		PHIV with NA (5)	10			Verbal IQ = 80.5 vs. 65.6 vs. 78.4
		HIV− (40)	11.84			
Puthanakit et al. (2013)	Thailand and Cambodia	HIV exposed (155)	7	58% Low income	Wechsler intelligence scale	Full-scale IQ = 86 vs. 90
						Verbal IQ = 82 vs. 87
		HIV unexposed (164)	7	35% Low income		Performance IQ = 91 vs. 94
Weber et al. (2017)^***^	Germany	PHIV (14)	8.24	N/A	Wechsler intelligence scale	Full-scale IQ = 106.5
						Verbal IQ = 106

N/A, Not applicable; HEU, HIV-exposed uninfected; HUU, HIV-unexposed uninfected; PHIV, Perinatally HIV-infected; with or without NA, with or without neuroimaging abnormalities; HIV+, HIV-infected; HIV−, HIV negative;^*^Study without control group in which B-cell counts and percentages were strongly associated with poorer IQ; ^**^Study without control group in which it was found that poverty and longer duration of untreated HIV affects IQ scores; ^***^Study without control group in which neurocognitive performance correlated with early and sufficient viral load suppression within the first 3 years of life. The Wechsler intelligence scale helps to determine the global capacity of a person (child, adolescent, or adult) to act purposefully, to think rationally, and to deal effectively with his environment (Wechsler, 1940). The scale has many versions depending on whether children or adults are being tested, and subscales of the version can be found depending on the type of IQ they measure (Weiss et al., 2019). Referring to the table, we have included full-scale IQ (which evaluates an individual’s complete cognitive capacity, including *v*erbal comprehension index, perceptual reasoning index, and working memory index), verbal IQ (which measures verbal comprehension and working memory), and performance IQ (which measures perceptual organization and processing speed).

## HIV infection, neurons, and synaptic connections

Evidence presented by Yu et al. (2019) suggests that HIV delays cortical maturation, with cortical atrophy present in adolescents (gray matter volume is widely reduced in the frontal, temporal, and insular regions, and in the cerebellum bilaterally). Poor cortical development has therefore been identified as the key aspect responsible for cognitive impairment. As indicated in the previous section, genes associated with higher intelligence accumulate in cortical pyramidal neurons, which concurs with the observations of Yu et al. Soon after an individual is infected by HIV, the virus enters the central nervous system (CNS), and generates an encephalitis within a few days after the exposure event (Valcour et al., 2012). HIV infection, in the early stages, induces a CNS inflammatory T-cell response, with vasculitis and leptomeningitis, and directly damages oligodendrocytes, neurons, and white matter (Gray et al., 1996). Through this process, which is also observed in the cerebral cortex of AIDS patients, HIV induces neuronal apoptosis, which is a potential explanation for the diffuse poliodystrophy seen in the cerebrums of such cases (Ozdener, 2005; Soontornniyomkij, 2017). In these cases, it is assumed that HIV indirectly induces the destruction of neuronal cell bodies which are known to lie within the cerebral gray matter.

Aside from gray matter, it has also been demonstrated that HIV infection detrimentally affects brain white matter. In contrast to gray matter, white matter is largely made up of a fatty substance called myelin, which coats and protects neurons, and enables neurons to transmit signals more rapidly and efficiently. A reduction of the white matter in the brain of HIV-positive individuals has been extensively described in several publications (McMurtray et al., 2008; Mina et al., 2021; Sarma et al., 2021). Importantly, a reduction in white matter may be associated with motor and cognitive impairment (Filley et al., 2015; Peng et al., 2020). This has been suggested by researchers who have attempted to unravel the potential mechanisms whereby HIV infection blocks the maturation process of the brain cells that produce myelin (Roth et al., 2021). In relation to this, Roth et al. have observed that HIV-induced neuroinflammation inhibits the maturation of myelin-producing brain cells called oligodendrocytes via glutamate-dependent activation of the PKR-like ER kinase (PERK) arm of the integrated stress response (Roth et al., 2021). In the absence of oligodendrocyte maturation, HIV effectively inhibits white matter production.

It is acknowledged that HIV does not productively infect neurons, and the deleterious effects of HIV on neurons are largely indirect, essentially triggered by HIV neurotoxicity, which is mediated by a neuroinflammatory response to viral proteins and inflammatory cytokines released by infected microglia and macrophages (Kaul et al., 2001; Ellis et al., 2007; Saylor et al., 2016). Consequently, HIV infection can potentially cause the loss of excitatory synaptic connections in HIV-infected individuals. Studies which speculate on and reveal the potential mechanisms underlining this process have been extensively reviewed by Green et al. (2019). They have shown that while some studies indicate that synapse loss induced by HIV is an integral part of the agonal event intimately associated with neuronal destruction, other studies (using models in which discrete application of HIV neurotoxins mimic the early phase of infection) support a mechanism more similar to the process of homeostatic scaling. Fortuitously, the synaptic loss and the resulting cognitive impairment induced by HIV proteins has been observed to be pharmacologically reversable via use of a GluN2B antagonist drug called ifenprodil (Raybuck et al., 2017).

**Figure 2:**
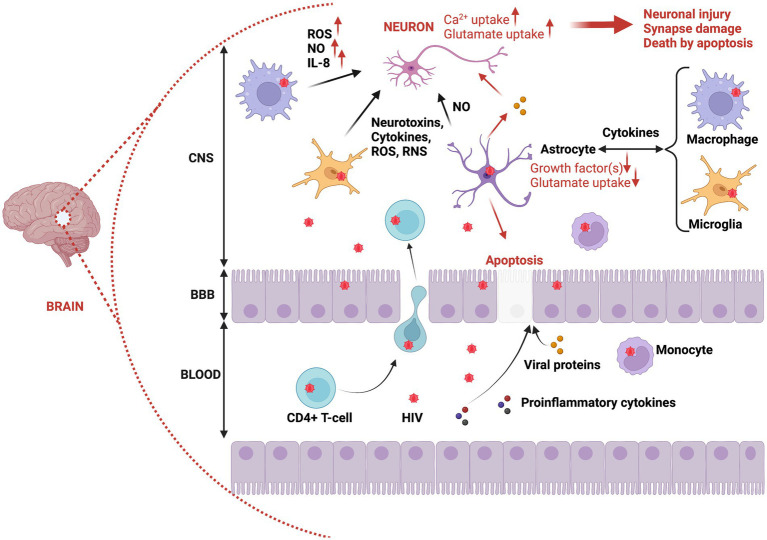
Brain invasion by HIV, and the immunopathogenesis of neuronal damage and death. HIV can overcome BBB protection of the brain via infected CD4+ T-cells or infected monocytes migrating from the bloodstream to the CNS. This putative process of brain inoculation by HIV is referred to as the trojan horse theory. In addition, infected epithelial cells can be used to transport HIV particles from blood to the CNS (transcytosis). Also, proinflammatory cytokines and proteins can facilitate HIV invasion by altering the permeability of epithelial cells within the blood–brain barrier. Once in the CNS, HIV can infect macrophages, glial cells (microglia), and astrocytes. Thus, reactive astrocytes can trigger apoptosis of epithelial cells through release of viral proteins such as Tat, which also detrimentally affects neurons (increased damage and neuronal death). The infection of macrophages, microglia, and astrocytes provokes their activation, resulting in the production of proinflammatory cytokines, neurotoxins, ROS, and RNS, all contributing to neuronal damage and induction of neuronal death via apoptosis. CNS, Central nervous system; BBB, Blood brain barrier; ROS, Reactive oxygen species; NO, Nitric oxide; RNS, Reactive nitrogen species.

**Figure 3:**
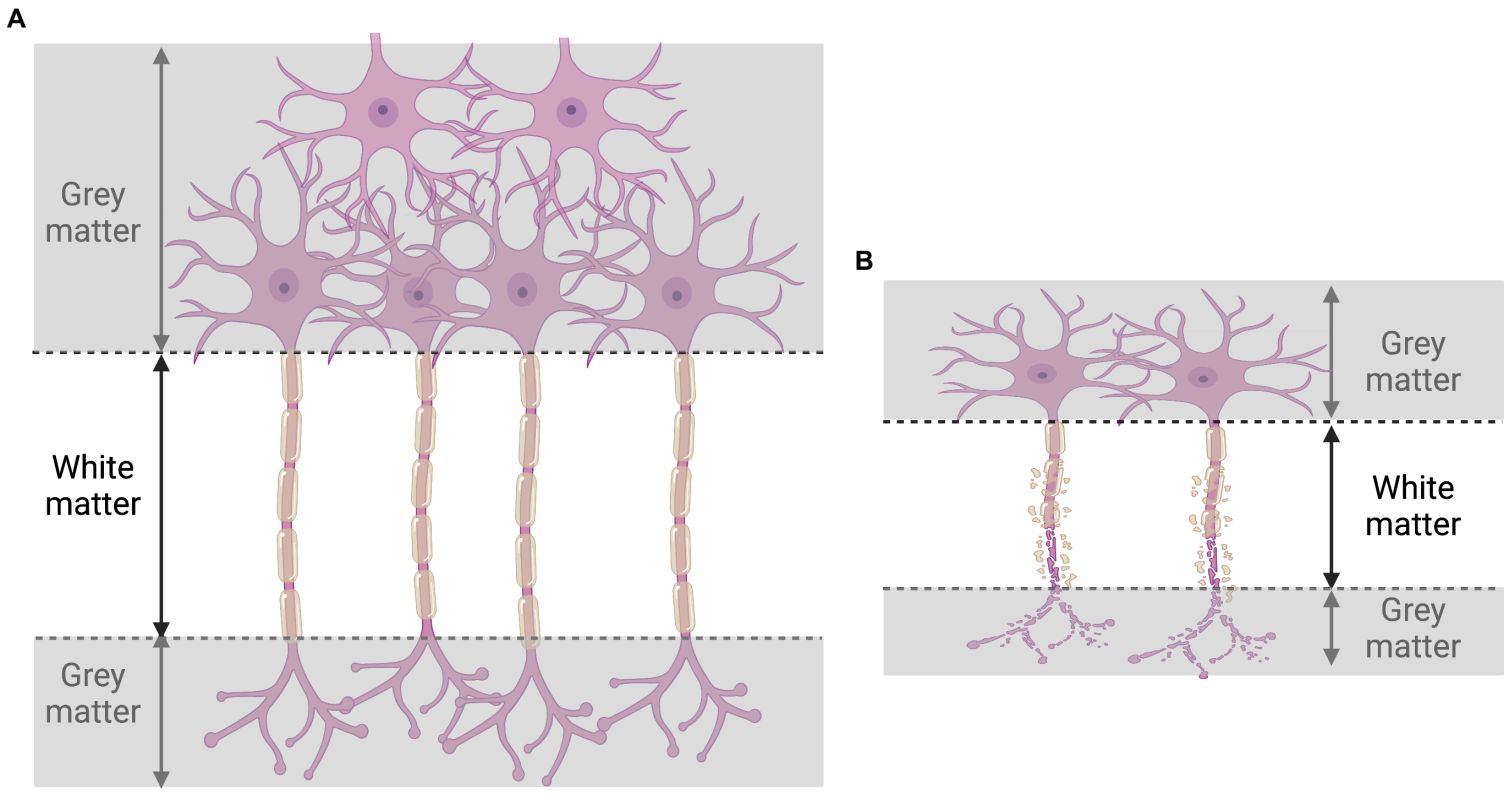
Illustration of consequences of HIV infection on gray and white matter. During HIV infection **(B)**, the destruction of neurons **(A)** provokes the loss of both gray and white matter.

A summary of mechanisms promoting negative effects of HIV on neurons is provided in Figures 2, 3.

## Intellectual impairment in HIV-positive individuals: evidence from perinatally infected children, HIV exposed children, and adults

Not many studies have reported on the relationship between HIV infection and IQ scores; however, one review of the contemporary literature (Table 1) has provided significant relevant information regarding the effect of HIV infection on IQ scores. Notably, nearly all studies reviewed were focused on children and adolescents. In general, this effect can be observed to be a negative association. Approximately 10 years ago, Martinez et al. (2012) observed that HIV-positive children display statistically significantly lower IQ scores (84.6) in comparison with HIV-negative children (91.7), particularly with respect to the verbal sub test (81.2 vs. 90.3, *p* = 0.05). Furthermore, these investigators observed that HIV-infected children at an advanced stage of HIV disease had lower IQ scores and measures of verbal performance than children with less-advanced HIV infection (*p* > 0.05). In light of the novelty of their findings, Martinez et al. assumed that further studies investigating their observations were warranted before definitive conclusions could be made. Two years later, Kerr et al. (2014) observed no significant difference in performance IQ scores [90.3 vs. 94.5; mean difference = −4.12; *p* = 0.06] between HIV-exposed uninfected children and HIV-unexposed-uninfected children in Thailand and Cambodia; however, HIV-exposed-uninfected children had lower overall scores. Interestingly, the verbal IQ scores [81.1 vs. 87.2; mean difference = −6.09; *p* = 0.002] and the full-scale IQ scores [85.2 vs. 90.3; mean difference = −5.07; *p* = 0.01] clearly show that HIV-exposed-uninfected children had lower IQ scores compared to HIV-unexposed-uninfected children. Although these observations potentially reveal the role that HIV plays in the neurodevelopmental outcomes of children, the findings particularly highlight HIV’s potential influence on the intelligence of children who are perinatally infected or exposed to HIV. In a similar manner, Cohen et al. (2015) have demonstrated that cognitive performance in HIV-infected children is poor compared to healthy socioeconomically matched controls. HIV-infected children (median CD4+ T-cell count = 770 × 10^6^ cells/L) scored lower than healthy controls in all cognitive domains, and particularly in intelligence, information processing speed, and attention/working memory. Specifically, the IQ score was calculated to be 76 (±15.7) in HIV-infected children vs. 87.5 (± 13.6) in healthy children (*p* = 0.002; Cohen et al., 2015). These investigators observed an 11 point lower total IQ score compared with controls, which is consistent with the general trend reported in previous studies from both industrialized (Nozyce et al., 2006; Puthanakit et al., 2010) and developing countries (Ruel et al., 2012; Boyede et al., 2013; Puthanakit et al., 2013). More recently (in 2019), it was reported that the IQ scores of children who had been perinatally infected with HIV (PHIV) were significantly lower when compared to healthy controls [81 (±11) vs. 97 (±15), *p* = 0.005] (Van den Hof et al., 2019). One meta-analysis, conducted by Musindo et al. (2022) reported that most published articles (between 2020 and 2022) observed significantly lower intellectual functions in children and adolescents living with HIV compared to their HIV-negative peers. In addition, Patel et al. (2019) have indicated that poverty and a longer duration of untreated HIV may predispose perinatally infected children to sub-optimal cognitive development, which is manifested by lower IQ scores.

Although the preliminary results from the study by Martinez et al. (2012) have been confirmed by other research teams over the years, a rational explanation for the mechanisms leading to such an outcome remains elusive. Nevertheless, researchers (Phillips N. et al., 2016; Alford and Vera, 2018) have reported that HIV infection is responsible for (i) cognitive impairment in the domains of working memory, executive function, and processing speed and (ii) deficits in visual memory and visual–spatial ability. Furthermore, Williams et al. have revealed that immune dysregulation during HIV infection is negatively associated with neurodevelopmental and neurocognitive performance in infants and children (Williams et al., 2021). Specifically, they have noted that markers related to monocyte activation [particularly CRP (Kapetanovic et al., 2010, 2014, 2021; Blokhuis et al., 2019; Hoare et al., 2020), IL-6 (Kapetanovic et al., 2010, 2014, 2021; Blokhuis et al., 2019; Sevenoaks et al., 2021), and sCD163 (Benki-Nugent et al., 2019; Blokhuis et al., 2019; Kapetanovic et al., 2021)] and inflammation are associated with reduced neurocognitive performance in HIV-infected and/or HIV-exposed-uninfected children. Thus, cognitive impairment specifically resulting from HIV infection may potentially explain the lower IQ observed in HIV-infected children. HIV may thus have the ability to infect and/or affect brain cells that are critical to the overt manifestation of human intelligence via mechanisms that are as yet not fully understood or, indeed, have not yet been identified. A subsequent pertinent question that thus arises is the question of which specific cells are being targeted? With certain knowledge that intelligence remains an enduringly stable attribute in an individual from young to old age, it is unfortunately likely that HIV-positive children afflicted with lower IQ scores will have to carry the burden of their relative cognitive deficiency throughout their adulthood.

Further investigations in HIV-infected adults vs. healthy controls with respect to intelligence are urgently warranted, as they could aid in further exploring IQ levels in patients who are not perinatally infected. Thus far, it has been demonstrated that HIV induces an intellectual impairment in newly diagnosed HIV-positive patients above 16 years of age (68% vs. 10% for the control group; *p* < 0.001; Sunmonu et al., 2015). Although there was a 1-year higher average education in the control group (HIV-negative adults), an entire set of tests (verbal and performance) using the adapted Wechsler Adult Intelligence Scale (WAIS) has shown that ART naïve HIV-positive adults attain lower scores of intelligence than HIV-negative adults (*p* ≤ 0.001; Sunmonu et al., 2015). Moreover, a large proportion of HIV-infected individuals had opportunistic infections (50%; Sunmonu et al., 2015), which could be key players influencing IQ scores too.

With respect to Table 1, it has been established that HIV influences the IQ levels of HIV-infected individuals, and as such, the potential influence of HIV-infection on the intelligence of people living with HIV may be seen as more than just speculation. However, an explanation solely relying on the influence of HIV infection on neurons, herein referred to as cells of intelligence, is patently incomplete. Indeed, research evidence reveals the role of the gut, and particularly the gut microbiota, on intelligence via its influence on brain development and function. As demonstrated by Oluwagbemigun et al. (2022) the composition of the gut microbiome has been related to intelligence scores. More specifically, the preceding authors have observed that people with a particularly dominant bacterial community, namely the Ruminococcaceae and Coriobacteriaceae-dominant community, have been observed to display significantly higher cognitive performance than those who do not. Interestingly, HIV infection is known to profoundly modulate gut integrity and the overall microbiome composition and diversity in HIV-infected individuals. Based on contemporary literature, we also herein present evidence that may potentially explain how the gut may influence human intelligence and how HIV infection, via its effects on the gut, may modulate human cognitive function and overall intelligence.

## The gut-brain axis

**Figure 4:**
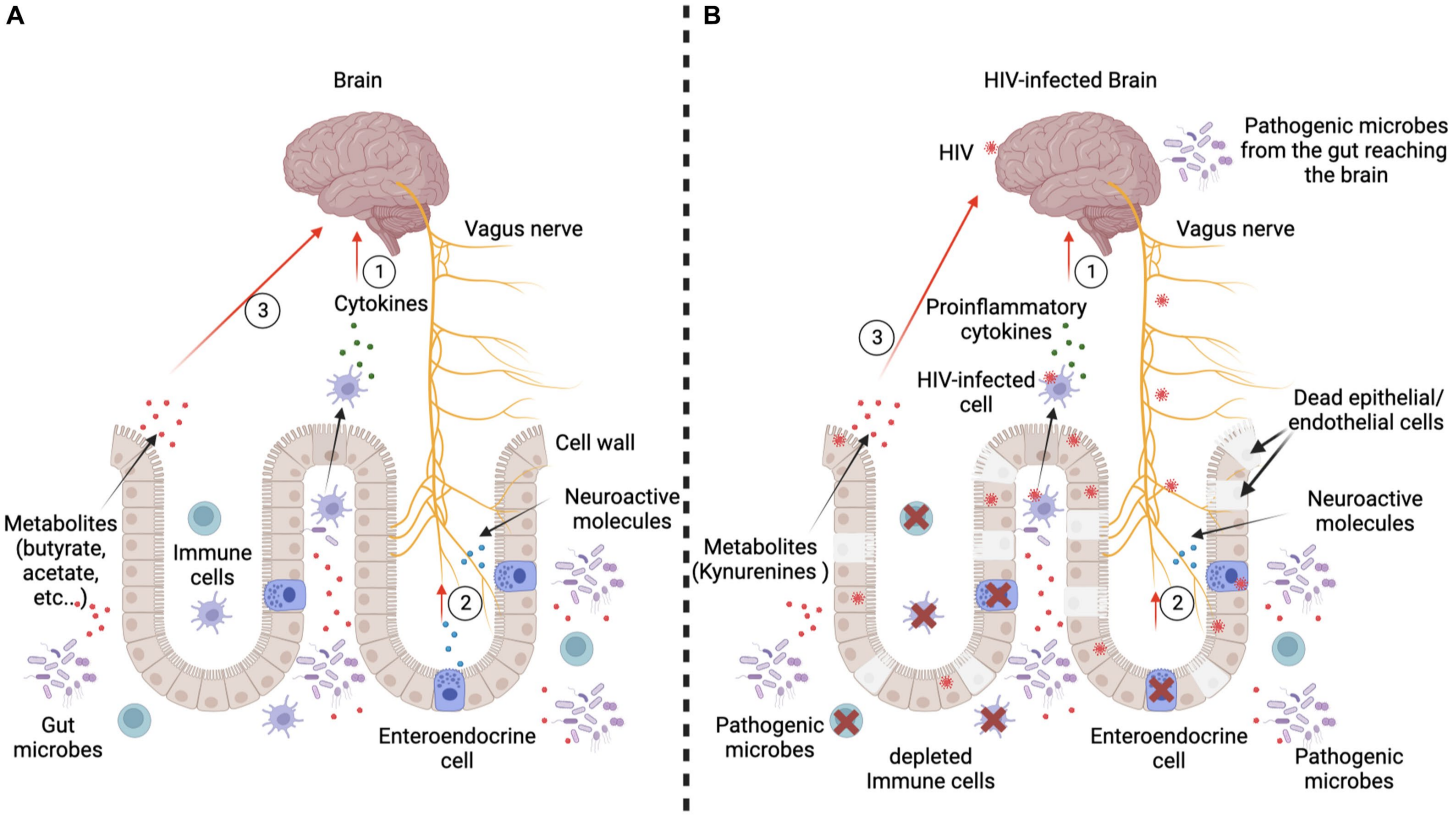
Passive and active mechanisms used by the gut to influence the brain. Panel **(A)** represents the scenario in an HIV-negative context. Three major communication mechanisms are present. First, immune cells interacting with the gut microbes produce cytokines which reach the brain through the blood circulation. They can either reduce or increase the inflammation, which influences the brain’s functioning (Haroon et al., 2014, 2016; Haroon and Miller, 2017). Second, gut microbes interact with gut cells, particularly the enteroendocrine cells, which produce neuroactive molecules (5-hydroxytryptamine/serotonin) and peptides (cholecystokinin, peptide YY, and glucagon-like peptide 1). Consequently, the vagus nerve is stimulated by these molecules and sends signals to the brain (Needham et al., 2020). Third, gut microbes produce metabolites (short-chain fatty acids like butyrate and acetate) and neurotransmitters (catecholamines, serotonin, gamma-aminobutyric acid), which are small enough to cross the blood–brain barrier to directly alter brain cell activity. Panel **(B)** represents the scenario in an HIV-positive context. Each of the major communication mechanisms reported in an HIV-negative context are profoundly altered. Indeed, the immune cells are depleted and/or produce proinflammatory cytokines. Thus, there is a proliferation of potentially pathogenic microbes which produce toxins that negatively affect brain development and/or functioning. HIV infection also causes abnormal functioning of the vagus nerve, which may induce gastric and intestinal slowing (slow transit time, resulting in constipation), and also results in an overgrowth of bacteria (George et al., 2014). Furthermore, during active HIV infection, gut epithelial/endothelial cells die or are sequestered and gut integrity is thus compromised, resulting in development of the leaky gut syndrome, which allows microbes to reach the brain via the bloodstream. Enteroendocrine cells are also depleted; hence, neuroactive molecules and peptides cannot efficiently modulate brain development and/or functioning within the CNS. Figure is adapted from Montiel-Castro et al. (2013).

It has been established that a bi-directional communication exists between the gastrointestinal tract (GI) and the CNS (Rhee et al., 2009). This complex interplay is referred to as the gut-brain axis, and reveals the extent of integration and crosstalk between the two critically important organs. As such, it is legitimate to speculate on the potential influence of the gut on human intelligence. Several key elements are involved in the complex interplay between the gut and the CNS. For example, the vagus nerve is well-known for playing roles in several important functions (Breit et al., 2018), including digestion, mood, and the immune response. Also, neurotransmitters (chemical messengers) and the gut microbiota, which is represented by an ecosystem of trillions of bacteria and other microorganisms, are also significantly important elements used to mediate and maintain the communication within the gut-brain axis. Thus, the gut and brain communicate via passive and active mechanisms including neural, immunological, and endocrine pathways (Shanahan, 1999; Drossman, 2005; Berthoud, 2008). Although the overall picture of these complex mechanisms remains unclear at present, the known pathways whereby the gut may influence brain activities are presented in Figure 4.

While Martinez et al. (2012) were demonstrating in their investigation that HIV infection influences intelligence quotient (IQ) scores of HIV-infected patients, Al-Asmakh et al. (2012) were substantiating in their study how the gut affects the developmental programming of the brain in the prenatal and postnatal contexts. Indeed, published data from epidemiological investigations have shown a possible association between neurodevelopmental disorders (e.g., autism and schizophrenia) and certain microbial pathogens (*Clostridium aminobutyricum, C. bifermentans, C. clostridioforme, C. difficile, C. colceatum, C. nexile, C. orbiscindens, C. ramosum, C. roseum, C. scindens*) which are responsible for infections located in the gut (Finegold et al., 2002; Mittal et al., 2008; Bale et al., 2010). Additionally, the presence of microbes such as Clostridia spores are believed to be responsible for the high rates of autism seen among siblings (Finegold, 2008). Experimental studies in rodents have revealed that exposure to some microbial pathogens (*Campylobacter jejuni, Escherichia coli*) during the prenatal period of life results in behavioral abnormalities, including anxiety and impaired cognition (Bilbo et al., 2005; Sullivan et al., 2006; Goehler et al., 2008). The fundamental reasons behind these outcomes remain to be clarified. However, since that the fetus develops in an almost sterile environment (Jiménez et al., 2005; Steel et al., 2005; Jiménez et al., 2008; Satokari et al., 2009), it is possible that maternal microbial metabolites may reach the growing fetus through the placenta, which is the main communication route between the mother and the fetus, and thus affect fetal brain development (Dancis, 1965). During the prenatal period, the precise underlying mechanisms whereby microbiota may influence brain development are yet to be determined; however, the placenta is believed to be one possible source. According to several publications (Alonso et al., 1991; Henry et al., 1994; Maccari et al., 1995; McCormick et al., 1995; Vallée et al., 1997; Caldji et al., 1998; Vallée et al., 1999), the placenta (also referred to as “fetal armor”; Kalb, 2012), (i) provides serotonin [essential hormone for fetal forebrain development (Bonnin et al., 2011)] to the fetus, (ii) sequesters its own tissues to nourish the brain of the fetus when the mother is deprived of food, (iii) may convert maternal tryptophan into the neurotransmitter, serotonin, and (iv) modulates the fetal hypothalamus-pituitary adrenal axis through hormonal interactions (via serotonin for example).

The postnatal period is also crucial for the brain development. Indeed, microbiota colonization of the GI tract, which is influenced by the mode of delivery (Dominguez-Bello et al., 2010) and feeding patterns (Pop, 2012), begins at birth. Clinical studies have demonstrated that breast-fed infants have more favorable neurodevelopment outcomes and higher IQ scores (Kramer et al., 2008; Guxens et al., 2011). Breast-fed infants appear to have more diverse and heterogenous gut microbiota compared to formula-fed infants, according to one metagenomic study by Schwartz et al. (2012). One interesting element of the composition of the gut microbiota is the influence of breastfeeding on the predominance of bifidobacteria. This association remains controversial, as some studies (Palmer et al., 2007; Adlerberth and Wold, 2009) have reported that there is no difference in bifidobacteria abundance between breast- and formula-fed infants. Nevertheless, the impact of bifidobacteria on brain development is worth mentioning. Indeed, bifidobacteria of human infant intestinal origin [predominantly *Bifidobacterium longum* and *Bifidobacterium bifidum* (Shkoporov et al., 2008)] in probiotics are suspected to have beneficial effects on depression in rats exposed to maternal separation stress in early life (Desbonnet et al., 2008). Furthermore, it has been demonstrated that bifidobacteria isolated from infant feces may influence brain fatty acid composition (Wall et al., 2012). Other than bifidobacteria, it is also known that the gut microbiota synthesizes vitamins K2 and B12, which are essential for human survival. *Lactobacillus reuteri* (Santos et al., 2008) is the primary producer of vitamin B12, which is essential for nervous system development (Dror and Allen, 2008; Marques et al., 2010). It can thus be confidently stated that gut microbiota can influence pre- and postnatal brain development.

The role of gut microbiota in the modulation of brain function in general, and of mood and/or behavior in particular during the lifetime has been the focus of many research studies (Bercik et al., 2011; Diaz Heijtz et al., 2011; Neufeld et al., 2011). Twenty years ago, Diaz Heijtz et al. (2011) postulated that the microbial colonization process initiates signaling mechanisms that may affect neuronal circuits involved in motor control and anxiety behavior. For example, in addition to the bacteria mentioned in the preceding discussion, infections by a fungus such as *Candida albicans* have been shown to be responsible for memory problems and brain abnormalities that resemble characteristics of Alzheimer’s disease. Indeed, Wu et al. (2019) have observed that when disseminated in the circulating blood, *C. albicans* can enter the brain, trigger inflammatory responses, and impair memory. *Candida albicans*, once in the brain, activates microglial and astroglial cells which aggregate around it, forming fungal-induced glial granulomas. Within the periphery of these granulomas and around the yeast cells, there are accumulations of amyloid precursor protein and cleaved amyloid beta (Aβ) peptides, respectively. In addition to Aβ peptides, the central nervous system, when infected by *C. albicans*, activates brain cells to produce the transcription factor NF-kB, and induce production of interleukin (IL)-1β, IL-6, and tumor necrosis factor (TNF), mainly to enhance phagocytic and antifungal activity by microglial cells. The presence of *C. albicans* in the brain, and the resulting inflammation, have been associated with memory impairment, which is reversed by fungal clearance (Wu et al., 2019). Although the preceding observations were seen in mice, the results may well be able to be extrapolated to humans, especially in a context where *C. albicans,* a commensal colonizer of the human gut, provokes disseminated infection, as in advanced HIV/AIDS. Thus, in a milieu of chronic dysbiosis, such as in HIV infection, where *C. albicans* is disseminated in the blood, memory impairment may be observed in humans. Our team (Zaongo et al., 2023) has previously postulated that during HIV infection, *C. albicans* may foster gut dysbiosis and easily disseminate throughout the body, including relocating to within other organs such as the brain.

To clearly understand how the gut affects the brain and potentially affects intelligence, it is critical to understand the key players in this process, and the mechanisms whereby they exert their influence. In Table 2, we summarize this key information, as reported in contemporary literature.

**Table 2:** The gut influences brain development and functioning via key elements.

Groups	Specific elements	Utility/reported effect	References
Metabolites or neurotransmitters	Serotonin	At the pre-natal level, is essential for fetal forebrain development	Bonnin et al. (2011)
	Vitamins K2 and B12	Essential for human survival and nervous system development	Dror and Allen (2008), Santos et al. (2008), and Marques et al. (2010)
	Acetate	Regulates microglial functions	Erny et al. (2021)
	Butyrate	Influences brain functions as it may regulate gene expression in the brain	Bourassa et al. (2016), and Alpino et al. (2022)
	Kynurenines (kynurenic acid, 3-hydroxykynurenine, quinolinic acid, and 3-hydroxyanthranilate)	They target neurotransmitter receptors and affect redox processes, and thus influence brain physiology	Schwarcz et al. (2012)
	Lipopolysaccharides	Induces anxiety or depressive-like or sickness behavior (fatigue, anorexia, low mood, or apathy later in life)	DellaGioia and Hannestad (2010), and On Wah et al. (2019)
Bacteria	*Lactobacillus reuteri*	Produces vitamin B12, which is essential for nervous system development	Dror and Allen (2008), and Marques et al. (2010)
	*Clostridium aminobutyricum C. bifermentans C. clostridioforme C. difficile C. colceatum C. nexile C. orbiscindens C. ramosum C. roseum C. scindens*	Responsible for infections located in the gut. These infections are linked to autism and schizophrenia as they influence the developmental programming of the brain	Finegold et al. (2002), Finegold (2008), Mittal et al. (2008), and Bale et al. (2010)
	*Campylobacter jejuni*	Induces behavioral abnormalities including anxiety and impaired cognition	Bilbo et al. (2005), Sullivan et al. (2006), and Goehler et al. (2008)
	*Escherichia coli*	Induces behavioral abnormalities including anxiety and impaired cognition	Bilbo et al. (2005), Sullivan et al. (2006), and Goehler et al. (2008)
	Bifidobacteria	May influence brain fatty acid composition	Wall et al. (2012)
		In probiotics (predominantly *Bifidobacterium longum* and *Bifidobacterium bifidum*), they are suspected to have beneficial effects on depression in rats exposed to maternal separation stress in early life	Shkoporov et al. (2008)
	*Slackia*	Produces equol, which is essential in maintaining homeostasis	Schröder et al. (2013), and Freedman et al. (2018)
	*A. muciniphila*	*May produce Serotonin*	Valles-Colomer et al. (2019)
		*Produces acetate which has beneficial effects on neurodegenerative conditions*	Mirzaei et al. (2021)
		*Produces propionate, which has beneficial effects on neurodegenerative conditions*	Mirzaei et al. (2021)
		*Reduces inflammation by producing indole and indole acetic acid from tryptophan metabolism*	Schröder et al. (2013), Freedman et al. (2018), and Liu et al. (2020)
	*Eubacterium hallii group*	*Produces propionate which has beneficial effects on neurodegenerative conditions*	Durack and Lynch (2019)
		Produces butyrate	Liu et al. (2020)
	*Lactococcus*	Influences levels of serotonin (modulates serotonin signaling/metabolism)	Oluwagbemigun et al. (2022)
		Produces histamine which is essential to regulate cognitive functions	Landete et al. (2008), and Thomas et al. (2012)
		Production of dopamine which is essential to regulate cognitive functions	Tetz et al. (2018)
	*Pseudomonas*	Influences levels of serotonin (modulates serotonin signaling/metabolism)	Oluwagbemigun et al. (2022)
		Production of gamma aminobutyric acid, a marker of Alzheimer’s disease	Manyevitch et al. (2018), and Strandwitz et al. (2019)
Fungi	*Candida albicans*	Memory impairment	Wu et al. (2019)
	*Geotrichum capitatum*	Can disseminate in lung, liver, skin. Thus, it may potentially infect the brain and influence its functioning	Gao et al. (2015)
	*Saccharomyces boulardii*	improves the behavior and emotions	Tao et al. (2021)
Virus	HIV	HIV induces neuronal apoptosis	Ozdener (2005), and Soontornniyomkij (2017)
		Induces permanent gut dysbiosis syndrome which allows translocation of microbial products in blood. Once in contact with the brain, they influence its functioning. Could influence brain function by killing enteroendocrine cells within the gut	van Marle et al. (2013), and Rich et al. (2020)
		Chronic inflammation whereby cytokines produced by immune cells can influence the brain functioning	Maek et al. (2007), Benakis et al. (2016), McArthur and Johnson (2020), and Salvo-Romero et al. (2020)
Enteroendocrine cells	EC cells, D cells, I cells, K cells, L cells	Produces neuroactive molecules (5-hydroxytryptamine/ serotonin) and peptides (cholecystokinin, peptide YY, and glucagon-like peptide 1) which modulate brain functions via enteric nervous system, vagus nerve, and spinal afferent fibers	Latorre et al. (2016), and Needham et al. (2020)
Immune cells (CD4+ T-cells, dendritic cells, macrophages, etc.)	Pro-inflammatory cytokines (IL-1, IL-17, IFN gamma)	Modulates brain development and functions via their receptors located in the hippocampus	Salvo-Romero et al. (2020)

## HIV infection negatively influences the gut-brain axis

It has been shown that virally suppressed HIV-infected individuals have higher levels of HIV DNA in the gut than in the blood (Chun et al., 2008; Yukl et al., 2013), suggesting the preeminent role of the gut in HIV immunopathogenesis. Indeed, it has been reported that the gastrointestinal tract (GI) represents one of the primary sites of HIV replication and reservoir survival (Wong and Yukl, 2016; Yoder et al., 2017). Some researchers believe that the gut is a vital site for ongoing/persistent HIV replication (Brenchley et al., 2004), particularly because the gut is the anatomical site with the highest concentration of CD4+ T-cells in the body. Aligned with this, many research teams have demonstrated that HIV infection modulates the gut microbiome (Ceccarelli et al., 2019; Rocafort et al., 2019;Vujkovic-Cvijin and Somsouk, 2019; Ouyang et al., 2020), which results in drastic changes in microbial diversity within the gut. Additionally, structural damage to the intestinal barrier, impairment of the mucosal immunological function, augmentation of microbial translocation, and long-term immune activation are well-documented consequences of HIV infection within the gut.

Compared to HIV-uninfected individuals, it has been shown that microbial communities (Vujkovic-Cvijin and Somsouk, 2019; Ouyang et al., 2020) and their metabolites (Dillon et al., 2017; Serrano-Villar et al., 2017) are profoundly modified during HIV infection. Within the gut, HIV infection has been associated to a reduction in symbiotic beneficial bacteria, and an increase in potentially pathogenic bacteria. In other words, beneficial bacteria like *Akkermansia muciniphila*, *Bacteroides*, *Faecalis*, *Bacteroides vulvae*, *Diplococcus*, and *Arbuscular Roseus* are reduced, while potentially pathogenic species such as *Proteus*, *Enterococcus, Klebsiella*, *Shigella*, and *Streptococcus* (Zhou et al., 2018; Geng et al., 2020) are enriched. *Akkermansia muciniphila*, for example, is possibly able to produce serotonin (Valles-Colomer et al., 2019), which is a key neurotransmitter that is essential to brain development and functioning, as shown above. In addition, *A. muciniphila* produces short chain fatty acids (SCFA) such as acetate and propionate (Mirzaei et al., 2021) from carbohydrate metabolism. These SCFA, including butyrate (produced by SCFA-producing bacteria which are significantly reduced after HIV infection), are considered to have a beneficial influence on many neurodegenerative conditions (Liu et al., 2020). *Akkermansia muciniphila* is also known for its ability to produce indole and indole acetic acid from tryptophan metabolism (Kaur et al., 2019). During HIV infection tryptophan in the gut is metabolized by bacteria utilizing the kynurenine pathway, which results in the production and accumulation of 3-hydroxyanthranilate [a subproduct of the kynurenine pathway (Serrano-Villar et al., 2017)]. This leads to intestinal barrier dysfunction, intestinal immune imbalance, inflammation (Vujkovic-Cvijin and Somsouk, 2019), and ultimately a perturbation of the gut-brain axis. Thus, *A. muciniphila*, as can be seen in the preceding discussion, participates significantly in the preservation and maintenance of the integrity of the gut-brain axis. In an HIV-infected context, it may well be seen (upon further research) that the reduction of *A. muciniphila* and its beneficial metabolites in the gut represents a potential cause for cognitive and overall intelligence perturbations. However, a reduction of *A. muciniphila* during HIV infection has not been established as a validated fact. In fact, there remains no accepted consensus on the precise profile of all beneficial gut bacteria. Further investigations are therefore required in this regard. It is, however, important to note that Oluwagbemigun et al. (2022) have observed that *Akkermansia* and other bacteria (*Ruminiclostridium 5*, *Ruminococcaceae UCG-010*, *Coriobacteriaceae*, *Slackia*, the *Eubacterium hallii group*, *Peptoclostridium*, *Lactococcus*, *Erysipelotrichaceae incertae sedis*, *Eubacterium nodatum group*, *Prevotellaceae*, *Robiginitalea*, *Pseudomonas*, and the *Bacteroidales S24-7 group*) are associated with fluid intelligence, which in turn, according to van Aken et al. (2016), correlates with cognitive performance. In the absence of a definitive list of bacteria influencing human intelligence, the list provided by Oluwagbemigun et al. (2022) is informative, and represent an important platform from which researchers may launch further investigations, particularly in an HIV infection-related context.

Gut integrity is known to be compromised during HIV infection (Ouyang et al., 2021; Zaongo et al., 2022b). Indeed, the combined effects of (i) immune cell depletion (Kløverpris et al., 2016) and (ii) the reduction of beneficial bacteria which trigger the production of B defensins and LL-37 by epithelial/endothelial cells promoting competition for adhesion sites (via secretion of Iald and SCFAs), considerably reduces the ability of the body to control the proliferation of *C. albicans* and other potentially pathogenic microorganisms (Zaongo et al., 2023). Consequently, the epithelial/endothelial cells within the gut of an HIV-positive individual are exposed to pathogenic microorganisms (Luo et al., 2019; Zaongo et al., 2023), toxins (Luo et al., 2019; Ferrari et al., 2022), biofilms, and hyphal forms (mainly from fungi; Ponde et al., 2021), which lead to the destruction of gut epithelial/endothelial cells. Moreover, HIV replication within intestinal cells provokes their death via apoptosis (mucosal apoptosis; Somsouk et al., 2015). Collectively, the preceding mechanisms induce the loss of gut integrity and cause a permanent leaky gut syndrome, promoting microbial translocation. Therefore, HIV can directly cause gastrointestinal dysfunction (also referred to as HIV enteropathy), which is also characterized by alteration of enteroendocrine cells (van Marle et al., 2013). Indeed, van Marle et al. (2013) have observed that HIV infection results in lower numbers of serotonin- and stomatostatin-immunoreactive cell counts in the gut tissues of HIV positive patients. Interestingly, they found that the preceding feature and high HIV viral loads are associated with survival in HIV-infected patients. This suggests that HIV provokes a reduction of enteroendocrine cells in the gut; however, the direct effect of this immunopathogenic mechanism on the overall intelligence of HIV-positive patients remains to be investigated. Our team is of the opinion that the loss of enteroendocrine cells may partially explain low IQ scores observed during HIV infection. Indeed, since enteroendocrine cells produce neuroactive molecules (5-hydroxytryptamine/serotonin) and peptides (cholecystokinin, peptide YY, and glucagon-like peptide 1), their depletion will significantly impact the production of previously mentioned neuroactive molecules, and ultimately the functions of the brain (Needham et al., 2020). With the knowledge that several types of enteroendocrine cells exist, future investigations will also need to consider the effects, during HIV infection, of each of these cells on intelligence.

It is well known that HIV infection induces immune cell depletion in general, and also that immune cells lose functional capacity in lieu of the chronic inflammatory state induced by HIV infection (Baxter et al., 2016; Vidya Vijayan et al., 2017; Nguyen et al., 2019; Wang S. et al., 2020; Le Hingrat et al., 2021). However, from results of studies conducted on animal models (Zonis et al., 2015; Chesnokova et al., 2016), it is also known that the immune system is critical for the development of behavioral and cognitive functions. As such, long-term illness and chronic intestinal inflammation have been shown in animal studies to be associated with behavioral disturbances such as cognitive impairment, impaired memory, depression, and anxiety. All of these conditions were observed to be linked to disrupted adult hippocampal neurogenesis. Therefore, the listed outcomes may be positively linked to immune system dysfunction, immune cell depletion, and the chronic inflammation seen during HIV infection. Furthermore, several studies have shown that pro-inflammatory cytokines are important mediators of abnormal brain development, particularly during perinatal infection, and as such, behavioral alterations following HIV infection may occur (Cunningham and De Souza, 1993; Cai et al., 2000; Urakubo et al., 2001). This may be explained by the presence of cytokine receptors (IL-1β for example), which are widely expressed in the hippocampus (Salvo-Romero et al., 2020). In addition, IL-17 has been found to be implicated in the pathogenesis of ischemic stroke (Iadecola and Anrather, 2011), suggesting the ability of proinflammatory cytokines to modulate brain functions. Benakis et al. have demonstrated that a reduction of effector IL-17 and gamma delta T-cells through commensal gut microbiota mitigates stroke outcomes (Benakis et al., 2016). In other words, the gut microbiota regulates cytokine expression, which in turns affects brain function. Therefore, in an HIV context associated with pathogenic microbes (where pathogenic microbes numbers are augmented in the gut), the persistent expression of pro-inflammatory cytokines such as IL-1 (Corley, 2000) and IL-17 (Maek et al., 2007; which significantly increase during HIV infection) for example, may be seen as being responsible for abnormal brain development and brain dysfunction.

Each of the preceding points may influence the gut-brain axis in an HIV-infected individual, and potentially influence cognition and overall intelligence.

## Perspectives

A more robust assessment of the influence of HIV infection on an individual’s intelligence requires an appreciable improvement of the overall quality of future studies into this fascinating realm of HIV research. As such, a diligent monitoring of the duration of HIV infection before ART initiation, particularly valid data concerning *in utero* exposure, duration, and ART regimen used would likely provide relevant information regarding the influence of ART on the IQ score of HIV-infected individuals. Furthermore, accurate records regarding premature birth or not, nutritional status, and IQ scores (resulting from extensive cognitive testing) before and after HIV infection are necessary to understand the direct effects of HIV infection on the intelligence of HIV-infected individuals. Also, an early brain assessment plus at least an annual MRI scan of the brains of different categories of HIV-infected individuals (some categories include newly diagnosed or treatment experienced; infants, children or adults; born to an HIV-infected mother or not) would help to assess structural alterations in the brain as HIV disease progresses, and ultimately physically study the particular neuron clusters and neurons (types, genes expressed) located at each of the altered sections of the brain. All of the preceding recommendations should help significantly in ensuring valid and robust data for these studies. Furthermore, identification of potential biological markers associated with intelligence deficiency during HIV infection would be an extremely useful clinical tool in the investigative armamentarium. Identification of potential confounding factors which may otherwise distort our clear understanding of the influence of HIV infection on the cognitive machinations of HIV-positive individuals is also mandatory. Finally, a standardized approach to research endeavors into HIV-related cognitive studies should be developed to ensure the comparability and consistency of investigations (*in vivo*, *in vitro*, or post-mortem), and to create a clearer picture of the potential associations between HIV infection and human cognition and intelligence.

Although the neuron can be considered as an integral and fundamental cellular component of intelligence, a specific focus on pyramidal neurons is required, especially in relation to HIV infection. The effect of HIV infection on CA1 pyramidal neurons has been evaluated in HIV transgenic rats, and results indicate that HIV infection reduces the excitability of CA1 pyramidal neurons (Sokolova et al., 2019). The authors of the preceding study further mention that this outcome is likely to disrupt neuronal network function and contribute to the development of HAND and depression. We believe that further investigations in a clinical setting could expose a potential association between the reduced excitability of pyramidal neurons and IQ scores. Thus, therapeutic approaches may be developed to potentially mitigate the emergence of intellectual deficiencies during HIV infection. Other than pyramidal neurons, other types or sub-types of neurons should be studied and identified. In this regard, depending on their gene signatures for instance, single cell sequencing could potentially be used to discriminate between different varieties of neurons in HIV-infected individuals. Perhaps it may be seen that some of these neurons present exhaustion signatures during HIV infection, which further favors their potential demise via apoptosis. HIV-positive individuals may thus achieve lower IQ scores compared to their HIV-negative counterparts via mechanisms such as this. Single cell sequencing is a powerful laboratory tool that is now being used to unravel some of the enigmas of HIV immunopathogenesis (Zaongo et al., 2022a), and as such, it may well be considered in future studies which delve into the subject of deciphering the influence of HIV on the intelligence of HIV-infected individuals.

In addition to focusing solely on the brain, analyses integrating the influence of the gut on the brain before and during HIV infection should be explored, despite the results of some past studies related to the influence of the gut on intelligence scores being inconsistent. Indeed, on the one hand, one study in the Netherlands conducted in older adults without any major health conditions, found no association between gut bacteria and thinking skills, or cognitive ability. On the other hand, Saji et al. (2019) have reported that patients with mild cognitive impairment without dementia had a higher prevalence of *Bacteroides* in their gut. Furthermore, they have indicated that patients with higher numbers of *Bacteroides* were more likely to present with white matter hyperintensity and cortical and hippocampal atrophy (potentially referring to Alzheimer disease). However, higher fractions of *Bacteroides* have also been linked to higher cognitive functioning, as reported by other teams (Carlson et al., 2018; Tamana et al., 2021). Besides, while Oluwagbemigun et al. (2022) have observed that Ruminococcaceae- and Coriobacteriaceae-dominant communities are associated with higher intelligence scores, some have reported that (i) Firmicutes-dominant and Bacteroidetes-dominant clusters (Tamana et al., 2021) are positively associated with higher cognitive functioning. In analyzing the age of the participants in the preceding studies (adults for some studies and infants for the other studies), one may legitimately believe that the relationship between gut microbiota composition and cognition and/or overall intelligence depends on age. In addition to age, a study by Kühn et al. (2019) has observed that sex, education level, average food intake, and tyrosine intake may likely modulate scores of intelligence. Indeed, they have observed that the preceding demographic and nutritional variables may account for approximately 6% of the variance in intelligence scores. This information suggests that diet, which is largely influenced by lifestyle in general, may induce changes to gut microbiota composition. Collectively, the preceding results confirm the notion that the exploration of the gut’s influence on intelligence is significantly difficult, requires mitigation of potential confounding factors (age, sex, body mass index, food intake, antibiotics, antifungal, or antiviral therapy, etc.), and requires standardized investigative approaches to facilitate the comparability of findings. Thus, further future studies (i) with larger study populations and (ii) incorporating cognitive and performance tests, and brain MRI performed at the same time point as stool collection, should be conducted. Moreover, studies integrating stool collection and exploration of the gut microbiome composition cognizant of the perspectives mentioned above (1st and 2nd paragraphs) will significantly assist in the understanding of the mechanisms whereby HIV infection may affect the overall intelligence of HIV-infected individuals. Lastly, the presence of comorbidities [e.g., colorectal cancer (Coghill et al., 2018)] and/or opportunistic infections [e.g., candidiasis (Zaongo et al., 2023)] may have significant associations with the gut-brain axis, and therefore their potential influence on IQ scores and intelligence deserve to be investigated. As such, Lomelí-Martínez et al. (2022) have noted that, in general, oral infections are associated with HIV/AIDS patients. They usually manifest in an immunodeficiency context and, as such, we believe that they may have a negative association with IQ scores and overall intelligence. Indeed, immunodeficiency resulting from HIV infection is associated with depletion of key immune cells which participate in gut-brain axis interactions (mainly CD4+ T-cells) and persistent systemic inflammation (see Table 2). Thus, the depleted immune cells associated with persistent expression of pro-inflammatory cytokines may potentially result in a disturbance of gut-brain axis functioning. Furthermore, the oral cavity is an integral part of the gastrointestinal tract (which is a contiguous series of hollow organs joined in an extended, convoluted tube from the mouth to the anus, and as such, microorganisms responsible for oral infections may conceivably be further disseminated within the gut, where they potentially negatively influence gut functioning). For example, *C. albicans,* which is responsible for oral candidiasis, can disseminate from the mouth to within the upper and lower gut, and subsequently foster gut dysbiosis and systemic infection during HIV infection (Zaongo et al., 2023). We are of the opinion that HIV positive individuals having the preceding attributes are potentially likely to display lower IQ scores. Our opinion is further supported by the observations of some authors who have shown that oral colonization with microorganisms such as *Treponema denticola*, is associated with cognitive impairment (Pisani et al., 2022; Tang et al., 2022; Pisani et al., 2023). In a context of HIV infection, and when considering *C. albicans* as the microorganism of interest, the preceding outcome is potentially also likely. However, only future targeted investigations will provide definitive and indisputable answers to this proposed outcome.

Furthermore, a more lucid understanding of the effects of HIV infection on IQ scores may require clarification regarding the specific influence of each subtype of HIV (HIV-1 or HIV-2). Indeed, all of the studies presented in this review are based on investigations involving HIV-1. However, to date, no published study has reported the effects of HIV-2 or the effects of HIV-1 groups and/or subtypes on IQ scores. We believe that more aggressive HIV strains are likely to induce greater reductions in IQ scores. Thus, it is possible that on a level playing field, HIV-2 infected individuals display similar IQ scores compared to HIV-negative individuals and higher IQ scores compared to HIV-1 infected individuals. Finally, the cognitive trends within those affected by different groups and subtypes of HIV-1 require assessment and clarification in future investigations.

We believe that study of the impact of HIV on IQ is inextricably linked to study of the cognitive functional domains (working memory, executive function, etc.) and of the intelligence subdomains (fluid intelligence, crystallized intelligence). Indeed, as intelligence itself is difficult to define and possesses a large number of unknown variables, studies targeting the influence of HIV infection on the preceding domains and subdomains will facilitate easier and more targeted approaches to further investigation in this quite intriguing domain of scientific endeavor. Thus, studying the impact of HIV on IQ rather than individual cognitive functional domains, or intelligence subdomains, if possible, would establish the foundational groundwork with respect to a more comprehensive understanding of some of the unknown elements of human intelligence (particularly at the cellular level), and their potential interactions and relationships with HIV.

## Conclusion

In this article, we have presented contemporary evidence suggesting that HIV infection detrimentally influences an individual’s IQ score. It is well known that HIV is a neurotropic virus, and as such, the deleterious effects of HIV on neurons, which are also considered to be individual cells of intelligence, are likely to be the fundamental cause of IQ score deficiencies seen in HIV positive individuals. However, an explanation relying solely on the effects of HIV on the neuron is incomplete and inadequate. We have described how the gut can influence brain development and functioning via several pathways (immune cells, vagus nerve, microorganisms, metabolites, and neurotransmitters). Interestingly, several researchers have demonstrated that HIV infection profoundly alters the gut microbiota and the functional integrity of the gut microbiome. Thus, the negative influence of HIV infection on the gut-brain axis may also account for comparatively lower IQ scores seen in HIV positive individuals. While this may be true, a much broader comprehension of the effects of HIV infection on cognition requires further dedicated study, using contemporary and more robust investigational approaches (such as MRI scans and RNA-seq techniques). We have faith that the perspectives provided herein will inspire future studies into the compelling investigational realm of the influence of HIV infection on cognitive functioning.

## Author contributions

SZ: Conceptualization, Writing – original draft, Writing – review & editing. VH: Writing – review & editing. FR: Writing – review & editing. DD: Writing – review & editing. A-SO: Writing – review & editing. YC: Conceptualization, Funding acquisition, Writing – review & editing.
